# Effects of digital multimodal interventions on objectively measured physical activity in older adults: a systematic review and meta-analysis

**DOI:** 10.3389/fpubh.2026.1867281

**Published:** 2026-06-25

**Authors:** Mingshu Yin, Enliang Hu, Jiayi Yao

**Affiliations:** 1 Competitive Sports School Attached to Shenyang Institute of Physical Education, Shenyang, China; 2 College of Aviation and Sports, Civil Aviation Flight University of China, Deyang, China; 3 School of Physical Education, China University of Mining and Technology, Xuzhou, China

**Keywords:** digital health interventions, meta-analysis, objective monitoring, older adults, physical activity

## Abstract

**Background:**

With the acceleration of global population aging, low physical activity levels have become a primary threat to the health of older adults. Consequently, digital health interventions (DHIs) based on objective monitoring tools (e.g., accelerometers, wearable devices) are progressively emerging as a novel means to promote active health in older adults.

**Objective:**

This study aims to quantitatively evaluate the actual effects of DHIs based on objective monitoring data on the physical activity levels of older adults through a systematic review and meta-analysis.

**Methods:**

PubMed, Web of Science, Embase, The Cochrane Library, and CINAHL were searched up to April 12, 2026. Randomized controlled trials (RCTs) that utilized objective instruments, such as accelerometers or smart sensors, to record DHIs promoting physical activity in older adults were included. A random-effects model was used for the meta-analysis, with mean differences (MD) applied to evaluate effect sizes. The quality of the included studies was assessed using the Cochrane Risk of Bias tool (RoB 2.0). Sensitivity and subgroup analyses were performed to explore sources of heterogeneity, and a trim-and-fill method was implemented for stress testing. The certainty of the evidence was evaluated using the GRADE system.

**Results:**

Ultimately, 21 RCTs involving 2,962 participants were included. Meta-analysis results demonstrated that DHIs significantly increased daily steps in older adults [MD = 381.58, 95% CI (237.58, 525.58), *p* < 0.00001], and Egger’s test detected no significant publication bias (*p* = 0.062). In contrast, while the original pooled effect for moderate-to-vigorous physical activity (MVPA) duration was significant [MD = 34.78, *p* < 0.00001], si gnificant publication bias was present (*p* = 0.033). After adjusting and filling 6 studies using the trim-and-fill method, the effect size decreased to 19.70 and lost statistical significance (*p* = 0.059). Exploratory subgroup analysis indicated that wearable devices and remote human feedback exhibited a trend toward better effect sizes for daily steps, and the interventions tended to yield superior MVPA improvements in populations with specific health risks compared to generally healthy older adults; however, these findings possess only hypothesis-generating value. The GRADE evaluation revealed that the certainty of evidence was at a “Low” level for weekly MVPA duration and at a “Very Low” level for daily steps.

**Conclusion:**

Limited low-certainty evidence suggests that DHIs may have potential promotive benefits in increasing the total amount of daily activity in older adults, but their robustness in driving MVPA remains insufficient. Digital interventions exhibit relatively better behavioral promotive benefits when targeting populations with specific health risks, but they should not be considered a standalone means to improve exercise intensity in clinical promotion. Future research and development should explore transitioning from simple data recording to intelligent closed-loop systems that incorporate intensity regulation and multidimensional feedback, thereby enhancing the robustness of intervention effects.

**Systematic review registration:**

https://www.crd.york.ac.uk/PROSPERO/view/CRD420261366882, CRD420261366882.

## 1 Introduction

With the continuous acceleration of the global population aging process, the high incidence of chronic diseases and the decline of physical functions in older adults have become a severe global public health challenge (1). As a core intervention to maintain metabolic health, improve cardiorespiratory fitness, and delay cognitive decline in older adults, physical activity (PA) is universally recognized as the physiological cornerstone for achieving “active aging” (2, 3). However, the physical activity levels of the older adult population globally generally fail to meet the recommended standards of the World Health Organization (WHO) (4), and prolonged sedentary behavior directly leads to a series of negative health outcomes, such as falls, frailty, and diminished quality of life (5). Given the heavy socioeconomic burden imposed by physical inactivity and its significant threat to the healthy life expectancy of older adults (6, 7), developing and validating targeted behavioral intervention strategies to reverse this negative trend has become an urgent task in the current public health field (8–10).

Among various strategies to improve the physical activity levels of older adults, daily walking and resistance exercises are widely preferred as choices for community interventions due to their low entry barriers and high health benefits (11). However, traditional intervention modes frequently rely on the self-discipline of participants and retrospective follow-ups. However, traditional intervention models often rely on participants’ self-motivation and post-hoc follow-up, and face practical challenges in real-world implementation, including difficulties in quantifying physical activity levels (12), the absence of evidence-based guidance in home settings (13), and the inability to adjust intervention intensity in real time. These limitations result in considerable inter-individual variability in intervention outcomes and persistently low long-term adherence (14). In recent years, the emergence of Digital Health Interventions (DHIs) has provided the technological foundation for overcoming these barriers (15). The term “digital multimodal interventions” in the context of this study specifically refers to systematic behavioral remodeling programs that implement multidimensional combinations of technical media and diverse interaction mechanisms within the architecture of DHIs. To resolve the technical heterogeneity brought by diverse media, such as smart wristbands, mobile applications, web platforms, Zoom meetings, and virtual agents, this study operationalizes them uniformly within a two-dimensional conceptual framework encompassing the hardware carrier mode (interaction between standalone wearable hardware and mobile apps or web platforms) and the delivery subject mode (integration of automated technology-driven and remote human-assisted or technology-assisted types) (16, 17). Although the aforementioned interventions are highly heterogeneous in their hardware and software manifestations, their quantitative pooling is methodologically appropriate because their underlying theoretical mechanisms are fully homogenous, as they all center on dynamic data captured by objective sensors to trigger and remodel active health management behaviors in older adults by integrating diverse behavior change techniques (BCTs), such as goal setting and self-monitoring. By constructing a diverse closed loop of software-hardware synergy and human-computer interaction, digital multimodal interventions aim to overcome the singularity of a single technical medium in triggering behavioral promotion benefits, thereby providing a prerequisite pathway for precisely evaluating multidimensional physical activity levels in older adults (18).

Current evidence from systematic reviews has preliminary explored the potential of digital interventions in promoting health among older adults. Multiple studies indicate that through mobile health (mHealth) or remote monitoring measures, the self-management self-efficacy and functional physical performance of older adults can be improved (19). However, regarding the two core indicators of physical activity (daily steps and moderate-to-vigorous physical activity (MVPA)), the results of existing studies still exhibit significant inconsistencies. First, the confounding of measurement instruments compromises the veracity of the evidence (20). Most existing reviews frequently mix subjective recall scales with objective monitoring data. Due to variations in cognitive function and social desirability effects, older adults often exhibit significant recall bias in subjective reports (21), which can lead to a substantial misestimation of intervention effects. Second, the driving effect of digital interventions on activity intensity remains unclear. Existing studies mostly focus on the volume indicator of daily steps; as for MVPA duration, which holds greater clinical significance for preventing chronic diseases, whether digital feedback mechanisms can achieve the transition from “volume increase” to “intensity target attainment” (22) currently lacks systematic integration based on purely objective data (23). Furthermore, existing research has classified technical media based on the degree of digital intervention interaction (e.g., interactive, semi-interactive, and passive) to evaluate their impacts on specific physical activity and physiological outcomes in older adults (24). However, against this background, whether technical pathways with different interactive characteristics can synergistically and objectively drive long-term adaptation in daily activity volume (daily steps) and exercise intensity (MVPA) (25) remains unsystematically elucidated in academia (26). Which technical modes and population characteristics can generate high-certainty benefits remains a clear gap in the evidence chain of purely objectively measured data (27, 28).

This study quantitatively evaluates the specific effects of digital health interventions (DHIs) on objectively measured physical activity in older adults through a systematic review and meta-analysis. By strictly including randomized controlled trials (RCTs) based on objective monitoring methods, such as accelerometers and pedometers, this study aims to evade self-report bias and reveal the true effects of the interventions on behavior change. In addition, through subgroup analyses of intervention characteristics and population attributes, it provides scientific support for constructing precise digital exercise prescriptions for older adults.

## 2 Materials and methods

This systematic review protocol was registered on PROSPERO (Registration Number: CRD420261366882). The final implementation schema, definitions of outcome measures, and statistical analysis plans of this systematic review are entirely consistent with the design initially submitted to the platform, and no protocol deviations occurred. The implementation and reporting of this systematic review strictly adhere to the Preferred Reporting Items for Systematic Reviews and Meta-Analyses (PRISMA 2020) statement (29).

### 2.1 Search strategy

A systematic literature search was independently conducted by two researchers across the PubMed, Web of Science Core Collection, Embase, The Cochrane Library, and CINAHL databases. The search period spanned from the inception of each database up to April 12, 2026. The search language for this systematic review was restricted to publicly published English literature. The search strategy utilized a combination of subject headings (e.g., MeSH, Emtree) and free-text words (e.g., Title/Abstract, Tiab). Specifically, the strategy was constructed through the cross-combination of core terms across four dimensions via Boolean logic operators: (1) older population: including “Aged”[Mesh], “Frail Elderly”[Mesh], older adult*, older adult, senior*, geriatric*, aging, aging, late life; (2) digital health interventions: including “Telemedicine”[Mesh], “Mobile Applications”[Mesh], “Smartphone”[Mesh], “Wearable Electronic Devices”[Mesh], “Fitness Trackers”[Mesh], digital health, mHealth, eHealth, telehealth, mobile app*, wearable*, smartphone*, activity tracker*, web-based, text messag*; (3) physical activity and behavioral outcomes: including “Exercise”[Mesh], “Motor Activity”[Mesh], “Sedentary Behavior”[Mesh], “Walking”[Mesh], physical activity, exercise*, walking, daily steps, step count*, MVPA, sedentary, sitting time, active lifestyle; (4) objective monitoring tools: including “Monitoring, Physiologic”[Mesh], pedometer*, activity tracker*, actigraph*, accelerometer*, sensor*. Given that the inclusion criteria of this systematic review were strictly limited to peer-reviewed, independently published randomized controlled trials (RCTs), no additional supplementary searches were conducted for gray literature or specific clinical trial registration platforms. Furthermore, the researchers performed standardized manual backward citation searching on the reference lists of the ultimately included studies and related systematic reviews to supplement primary studies that might have been missed during the database search. The complete step-by-step grid-based search procedures and the corresponding results for each database are presented in detail in Supplementary Text 1.

### 2.2 Inclusion and exclusion criteria

According to the PICOS principle, the following criteria were established: Participants (P): older adults with a mean age of 60 years or older. Intervention (I): behavioral change intervention programs centered on digital multimodal technologies, such as mobile applications, wearable devices, smart sensors, or web-based platforms. Comparison (C): control groups receiving usual care, standardized health education, being placed on a waiting list, or receiving non-digital interventions. Outcomes (O): physical activity must be assessed using objective monitoring tools, such as ActiGraph accelerometers, activPAL sensors, or pedometers. The primary outcomes were daily steps and weekly moderate-to-vigorous physical activity (MVPA) duration. Study Design (S): peer-reviewed, published randomized controlled trials (RCTs). Exclusion criteria: (1) duplicate publications; (2) studies evaluating physical activity solely using self-reported questionnaires; (3) studies where raw data could not be obtained or effect size conversion was impossible.

### 2.3 Data collection process and data items

Literature screening and data extraction were performed independently by two researchers. An initial screening was conducted by reading titles and abstracts, followed by a full-text review to determine the final eligible literature, and any discrepancies were resolved through discussion or by seeking consensus from a third-party expert. Through calculation, the inter-rater reliability between the two researchers during the independent literature screening stage yielded a Cohen’s Kappa coefficient of 0.74. For the extraction of quantitative data to be pooled, this study uniformly adopted and prioritized the extraction of change-from-baseline scores for the core outcome measures; post-intervention values were objectively extracted only when change scores were unavailable. The extracted content encompassed basic systematic review information, participant characteristics (age, sex, health status), outcome measures (daily steps, MVPA), and key details of the objective monitoring digital health interventions (such as health conditions) and intervention parameters (duration, carrier mode, feedback mechanism). Regarding the choice of time points, this study uniformly and strictly extracted post-intervention data immediately following the end of the intervention across all primary studies. For primary studies that simultaneously reported both immediate post-intervention data and long-term follow-up data (such as 6 months or 12 months), this review did not extract the longest follow-up data, nor did it perform mean calculations across different time points. This setting was intentionally designed to strictly avoid chronological heterogeneity triggered by behavioral decay after the withdrawal of interventions, thereby precisely isolating the immediate behavioral remodeling effects of the digital multimodal interventions themselves. For studies with incomplete data, attempts were made to contact the corresponding authors to obtain the missing information. If unavailable, the standard deviations of the change-from-baseline values SD change missing in the primary studies were calculated and converted strictly according to the methods recommended by the Cochrane Handbook, with the correlation coefficient set to 0.5. If a primary study only reported medians and interquartile ranges (IQR), these values were objectively converted into means and standard deviations required for meta-analysis according to standard statistical formulas (30).

### 2.4 Risk of Bias and certainty of evidence

The Risk of Bias tool 2.0 (RoB 2) recommended by the Cochrane Collaboration was used to evaluate the quality of the included studies (31). The evaluation encompassed five domains: bias arising from the randomization process, bias due to deviations from intended interventions, bias due to missing outcome data, bias in measurement of the outcome, and bias in selection of the reported result. Each domain was judged as “low risk,” “some concerns,” or “high risk,” which ultimately led to an overall risk of bias determination for each study. The GRADE system was applied to evaluate the certainty of evidence (32). Downgrading evaluations were performed across five dimensions: risk of bias of the study design, inconsistency, indirectness, imprecision, and publication bias, classifying the certainty of evidence into four levels: high, moderate, low, or very low. Two reviewers independently completed the quality assessment, and any disagreement was resolved through discussion to reach a consensus, with a third-party expert consulted when necessary.

### 2.5 Data analysis

The statistical analysis in this study was primarily performed using Review Manager 5.4 and R software. Within the R software environment, the script programming for the meta-analysis mainly invoked the meta and metafor packages. The pooling of continuous variables was uniformly executed through the metacont function, specifying either a three-level or a classic matrix model within the random-effects framework; for indicators presenting significant publication bias, the trimfill function was invoked to perform the trim-and-fill stress test. Since daily steps (steps/day) and weekly moderate-to-vigorous physical activity duration (MVPA min/wk) focused on in this review were both continuous variables, and all included studies utilized objective monitoring tools, this study estimated the pooled effect sizes by calculating the mean difference (MD) and 95% confidence interval (CI). Regarding the assessment of statistical heterogeneity, the *I*^2^ test was used for qualitative and quantitative determination: *I*^2^ ≤ 25% indicated low heterogeneity, 25% < *I*^2^ < 50% indicated moderate heterogeneity, 50% < *I*^2^ < 75% indicated significant heterogeneity, and *I*^2^ ≥ 75% indicated high heterogeneity (33). Considering the inherent differences among older participants regarding health baselines, cultural backgrounds, and digital intervention environments (home-based vs. community-based), and to improve the robustness of the conclusions while conservatively estimating the effect sizes, a random-effects model was uniformly adopted for the pooled analysis of all outcome measures in this study.

For robustness validation and heterogeneity exploration, this study adopted a multi-tiered processing strategy. Sensitivity analysis utilized a leave-one-out method, observing the dynamic changes in pooled effect sizes and heterogeneity to identify influential data points that exerted a disproportionate impact on the results. To thoroughly explore the extrinsic technical heterogeneity arising from diverse media among the included studies and to clarify the independent contributions of different technical modes, this study pre-specified subgroup analyses across four dimensions: first, within the technical carrier dimension, comparing the differences in effects between standalone wearable devices and pure software or interactive web-based platforms; second, within the delivery agent dimension, examining the similarities and differences in intervention effects between technology-driven pure automated monitoring modes and remote human feedback-assisted interventions; third, regarding population characteristics, stratifying the participants into general healthy community groups and groups with specific health risks or clinical sub-healthy characteristics for comparison; fourth, within the intervention duration dimension, using 12 weeks as the threshold to differentiate the impacts of short-term versus medium-to-long-term interventions. Furthermore, to fully elucidate the potential regulatory role of control group designs on the heterogeneity of weekly moderate-to-vigorous physical activity (MVPA) duration, this study implemented an additional post-hoc analysis, namely “nature of the control group (active control vs. inactive control),” after examining the extracted data to ensure complete transparency of the methodological procedures. Regarding the assessment of publication bias, following the Cochrane Collaboration standards, when the number of primary studies included for a single outcome measure was k ≥ 10, a funnel plot was used for visual inspection alongside Egger’s linear regression test for quantitative evaluation. If a significant risk of publication bias was present, the trim and fill method was further utilized for stress testing to determine the stability of the original pooled results by simulating the changes in effect sizes after accounting for missing studies. All statistical significance levels were set at *p* < 0.05.

## 3 Results

### 3.1 Literature screening and study selection

**Figure 1:**
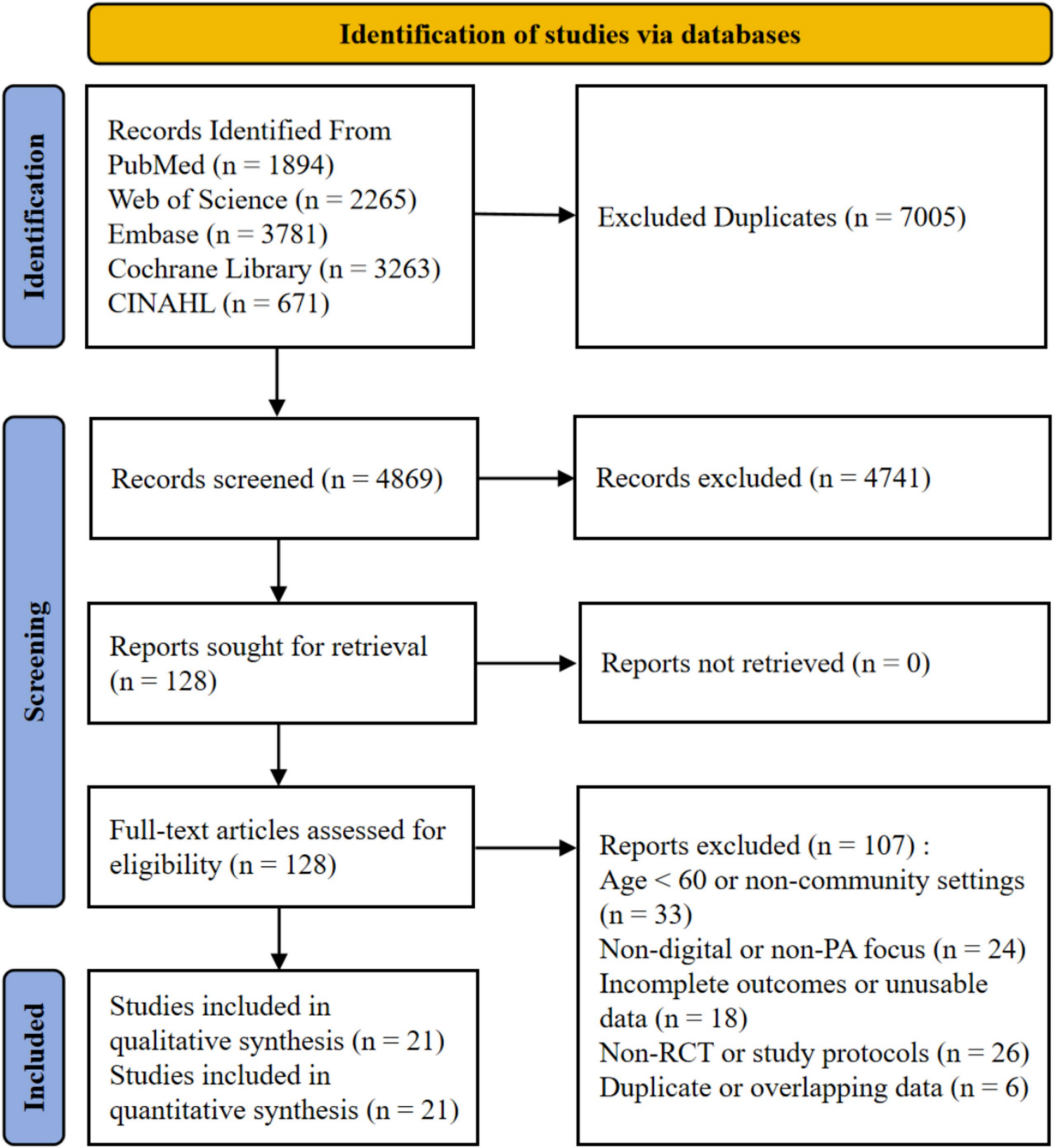
PRISMA 2020 flow diagram of literature search and screening for digital health interventions based on objective monitoring to promote physical activity in older adults.

The PRISMA 2020 flow diagram for the literature screening process in this study is illustrated in Figure 1. Through preliminary searches across the PubMed, Web of Science, Embase, Cochrane Library, and CINAHL databases, a total of 11,874 relevant records were identified. After removing 7,005 duplicate publications using reference management software supplemented by manual verification, the remaining 4,869 records entered the initial screening stage. During the title and abstract screening stage, a total of 4,741 studies were excluded because they did not meet the inclusion criteria. The primary reasons for exclusion included mismatched study populations (such as age non-compliance or non-community settings), non-digital interventions, outcome measures unrelated to objective physical activity, non-randomized controlled trial designs, and animal experiments. Following the initial screening, the researchers performed a rigorous full-text evaluation on the remaining 128 studies. According to the pre-specified PICOS criteria, an additional 107 studies were excluded for the following specific reasons: age less than 60 years or non-community living environments (*n* = 33), non-digital interventions or lack of focus on physical activity (*n* = 24), incomplete outcome measures or unavailable data (*n* = 18), non-RCT designs or study protocols only (*n* = 26), and duplicate or overlapping data (*n* = 6). Ultimately, 21 high-quality studies were included in this study for qualitative synthesis, and all included studies contained sufficient statistical information for quantitative synthesis (meta-analysis).

### 3.2 Description of included study characteristics

A total of 21 randomized controlled trials (RCTs) were included in this study, with publication dates spanning from 2009 to 2025 (34–54), among which studies published in 2020 and later accounted for more than 50% (41–54). The total sample size covered across the studies was 2,962 participants, with the sample size of individual studies fluctuating between 26 and 605 participants (52). The mean age of the participants was primarily concentrated between 60 (34) and 75 (51) years. Regarding population types, the study participants encompassed general community-dwelling older adults (39, 47, 49–52), insufficiently active older adults (35, 38, 46, 48), overweight or obese older adults (37, 42, 45), frail or pre-frail patients (44, 53, 54), and high-risk populations with diabetes or cardiovascular disease risks (34, 36, 40, 41, 43). Except for two studies that focused exclusively on female populations (36, 37), all remaining studies included both sexes, with the proportion of females generally ranging from 50 to 85%.

The digital platforms utilized across the studies exhibited diverse characteristics, primarily including mobile applications (Apps, such as WeChat, WhatsApp, LINE, Rehab-Fit) (41, 45, 47, 50, 53, 54), wearable devices (such as the Fitbit series, Jawbone, Pedometer) (34–40, 42–51), web-based online platforms, and virtual interactive technologies (such as Virtual Agent, Zoom) (35, 37, 38, 52). The intervention duration ranged from 4 weeks to 12 months, with 12 weeks (3 months) being the most common intervention period (41–43, 45–47, 50, 52). Within the theoretical frameworks, Social Cognitive Theory (SCT), self-efficacy theory, self-regulation theory, and the Behavior Change Wheel (BCW) were widely applied. The core behavior change techniques (BCTs) primarily concentrated on goal setting, self-monitoring, real-time or automated feedback, remote coaching or telephone counseling, and social support (34–54). Regarding control group settings, most studies utilized usual care, standard health education brochures, or waitlist controls, while some studies used basic pedometer monitoring or structured exercise advice as comparisons. For outcome measures, all included studies utilized objective monitoring tools. Specifically, 13 studies reported daily steps as a primary or secondary outcome measure (34, 35, 37, 38, 40–47), and 12 studies reported moderate-to-vigorous physical activity (MVPA) duration (36–39, 41, 44–46, 48, 52–54). ActiGraph accelerometers were most frequently used as measurement instruments (36–41, 44, 46, 48, 51–53), followed by activPAL monitors (42, 43), professional-grade pedometers (such as NL-800), or sensor data directly derived from smart wearable devices (34, 35, 45, 47, 49, 50). The follow-up periods of the studies mostly coincided with the end of the intervention, though some studies provided long-term tracking data from 3 to 12 months post-intervention. (Details are presented in Table 1).

**Table 1:** Basic characteristics of the included literature.

Study (Author/Year)	Int/FU Duration	Sample size (IG/CG Initial/Completed)	Age (Mean ± SD)/Sex (% Female)	Health status/population	Digital platform and theoretical framework	Core BCTs	Control Setting	Outcomes
Yates et al., 2009 (34)	12 months?12 months	33 + 31/34 (Init) 29 + 28/26 (Comp)	65 ± 8/34%	IGT (Impaired glucose tolerance)	Pedometer; Diabetes prevention/Self-efficacy theory	Education, personalized goals/steps, diary, action planning	Usual care (Brief)	Steps
Bickmore et al., 2013 (35)	12 months/12 months	263 (Init)	Mean 71.3/61%	Sedentary urban older adults	Tablet + Virtual Agent; ECA theory	Daily automated dialog, daily goal setting, automated coaching	Pedometer group	Steps
Ashe et al., 2015 (36)	6 months/6 months	13/13 (Init) 12/8 (Comp)	64.1 ± 4.5/100%	Sedentary older women	Fitbit One; SCT/Social Ecological Model	Goal setting, self-monitoring, feedback, social support, planning	Health education	MVPA
Cadmus-Bertram et al., 2015 (37)	16 weeks/N/A	25/26 (Init) 24/25 (Comp)	60.0 ± 5.2/100%	Overweight/obese sedentary older women	Fitbit One + Web; CALO-RE framework	Self-monitoring, goal setting, behavioral feedback, phone counseling	Usual care	MVPA
Muellmann et al., 2019 (38)	10 weeks/10 weeks	104 + 107/107 (Init) 85 + 93/90 (Comp)	68.6 ± 5.5/64%	Insufficiently active older adults	Web platform + Fitbit; Self-regulation theory	Goal setting, action/coping planning, feedback, self-monitoring	Waitlist control	Steps/MVPA
Oliveira et al., 2019 (39)	6 months/6 months	64/67 (Init) 54/57 (Comp)	71.1 ± 7.1/65%	General older adults	Fitbit/Pedometer; BCTs	Goal attainment scaling (GAS), telephone coaching, feedback	Standard health education	MVPA/Steps
Roberts et al., 2019 (40)	20 weeks/20 weeks	20/20 (Init) 18/19 (Comp)	72.0 ± 7.4/60%	CVD risk/High sedentary behavior	Fitbit Zip; Cognitive behavioral theory	Monitoring feedback, weekly SMS, goal setting	Structured exercise counseling	Steps
Kwan et al., 2020 (41)	12 weeks/13 weeks	16/17 (Init) 15/15 (Comp)	71.0(IQR9.0)/85%	Risk of cognitive decline	App + WhatsApp; Persuasive Technology	Personalized goals, monitoring, electronic reminders, video feedback	Routine behavior intervention	Steps/MVPA
Rosenberg et al., 2020 (42)	12 weeks/12 weeks	29/31 (Init) 29/25 (Comp)	68.4 ± 4.9/68%	Overweight/obese sedentary older adults	Jawbone handband; SCT/Habit formation	Telephone coaching, goal setting, environment reminders, self-monitoring	Healthy life handbook	Steps
Brickwood et al., 2021 (43)	12 weeks/12 months	37 + 38/42 (Init) 24 + 25/26 (Comp)	72.4 ± 6.1/64%	Risk of chronic diseases	Jawbone UP24 App; SCT theory	Goal setting, App/SMS feedback, self-monitoring	Usual care	Steps
Liu et al., 2021 (44)	14 weeks/3 months	22/18 (Init) 22/18 (Comp)	IG: 72.1 ± 3.7, CG: 80.4 ± 6.8/85%	Frail/Pre-frail older adults	Fitbit + App; BCTs	Goal setting, incentive badges, monitoring, action cues	Training + Lectures	Steps/MVPA
Zhou et al., 2021 (45)	12 weeks/N/A	40/41 (Init) 34/34 (Comp)	67.4 ± 5.1/66%	Overweight/obese older adults	App + Smartband; BCTs	Goal setting, diet/PA monitoring, feedback, WeChat social support	Pedometer hand manual	Steps/MVPA
Alley et al., 2022 (46)	12 weeks/24 weeks	78 + 96/69 (Init) 56 + 59/51 (Comp)	69.34 ± 4.32/78.6%	Insufficiently active older adults	Web-based + Fitbit; TPB/SCT	6 tailored suggestion modules, action planning, exercise video library	Waitlist control	MVPA/Steps
Cai et al., 2022 (47)	3 months/N/A	36/36 (Init) 34/30 (Comp)	66.9 ± 4.2/64%	Rural older adults	WeChat + Pedometer; SCT/HAPA model	Peer support, planning, micro-experience sharing	Standard health education	Steps
Pischke et al., 2022 (48)	9 months/9 months	242 (Init) 160 (Comp)	68.7 ± 5.4/62%	Insufficiently active older adults	Web/App + Fitbit; Self-regulation theory	Monitoring, goals, feedback, small group coaching, social support	Waitlist control	MVPA
Recio-R et al., 2022 (49)	3 months/3 months	81/79 (Init)	70.8 ± 4.0/61.3%	General older adults	App + Smartband; Behavior change theory	Lifestyle counseling, self-monitoring, dietary logs, automated feedback	Short behavior intervention	Steps
Kawaguchi et al., 2024 (50)	12 weeks/12 weeks	87/94 (Init) 80/85 (Comp)	IG: 69.9 ± 6.0, CG: 70.1 ± 6.7/53%	General older adults	ESP App + LINE; BCTs	GPS trajectory monitoring, rank-based rewards, educational columns	Basic monitoring (Google Fit)	Steps
Oliveira et al., 2024 (51)	12 months/12 months	290/315 (Init) 258/252 (Comp)	74.0 ± 8.0/70%	General older adults	Fitbit + Telephone; SDT theory	Risk assessment, goal setting, coaching, problem solving	Nutritional coaching	Steps
Uemura et al., 2024 (52)	12 weeks/36 weeks	15/14 (Init) 14/12 (Comp)	IG: 73.9 ± 3.9/CG: 69.4 ± 3.2/50%	General older adults	Zoom meeting; Self-directed/SCT	Exploratory tasks, small group discussion, goals, monitoring	Mail information push	MVPA
Lee et al., 2025 (53)	4 weeks/3 months	19/19 (Init) 16/18 (Comp)	71.8 ± 9.34/71%	Frail/Pre-frail older adults	Rehab-Fit App; Self-efficacy theory	Educational workshops, App guidance, video/audio feedback	Waitlist control	MVPA
Li et al., 2025 (55)	6 months/6 months	67/67 (Init) 52/54 (Comp)	69.3 ± 5.1/75%	Pre-frail older adults	mHealth App; LIFE model/BCW theory	Personalized plans, video guidance, remote monitoring, feedback	Health education	MVPA
Yates et al., 2009 (34)	12 months/12 months	33 + 31/34 (Init) 29 + 28/26 (Comp)	65 ± 8/34%	IGT (Impaired glucose tolerance)	Pedometer; Diabetes prevention/Self-efficacy theory	Education, personalized goals/steps, diary, action planning	Usual care (Brief)	Steps
Bickmore et al., (35)	12 months//12 months	263 (Init)	Mean 71.3/61%	Sedentary urban older adults	Tablet + Virtual Agent; ECA theory	Daily automated dialog, daily goal setting, automated coaching	Pedometer group	Steps
Ashe et al., 2015 (36)	6 months/6 months	13/13 (Init) 12/8 (Comp)	64.1 ± 4.5/100%	Sedentary older women	Fitbit One; SCT/Social Ecological Model	Goal setting, self-monitoring, feedback, social support, planning	Health education	MVPA
Cadmus-Bertram et al., 2015 (37)	16 weeks/N/A	25/26 (Init) 24/25 (Comp)	60.0 ± 5.2/100%	Overweight/obese sedentary older women	Fitbit one + Web; CALO-RE framework	Self-monitoring, goal setting, behavioral feedback, phone counseling	Usual care	MVPA
Muellmann et al., 2019 (38)	10 weeks/10 weeks	104 + 107/107 (Init) 85 + 93/90 (Comp)	68.6 ± 5.5/64%	Insufficiently active older adults	Web platform + Fitbit; Self-regulation theory	Goal setting, action/coping planning, feedback, self-monitoring	Waitlist control	Steps/MVPA

### 3.3 Risk of Bias evaluation of included studies

**Figure 2:**
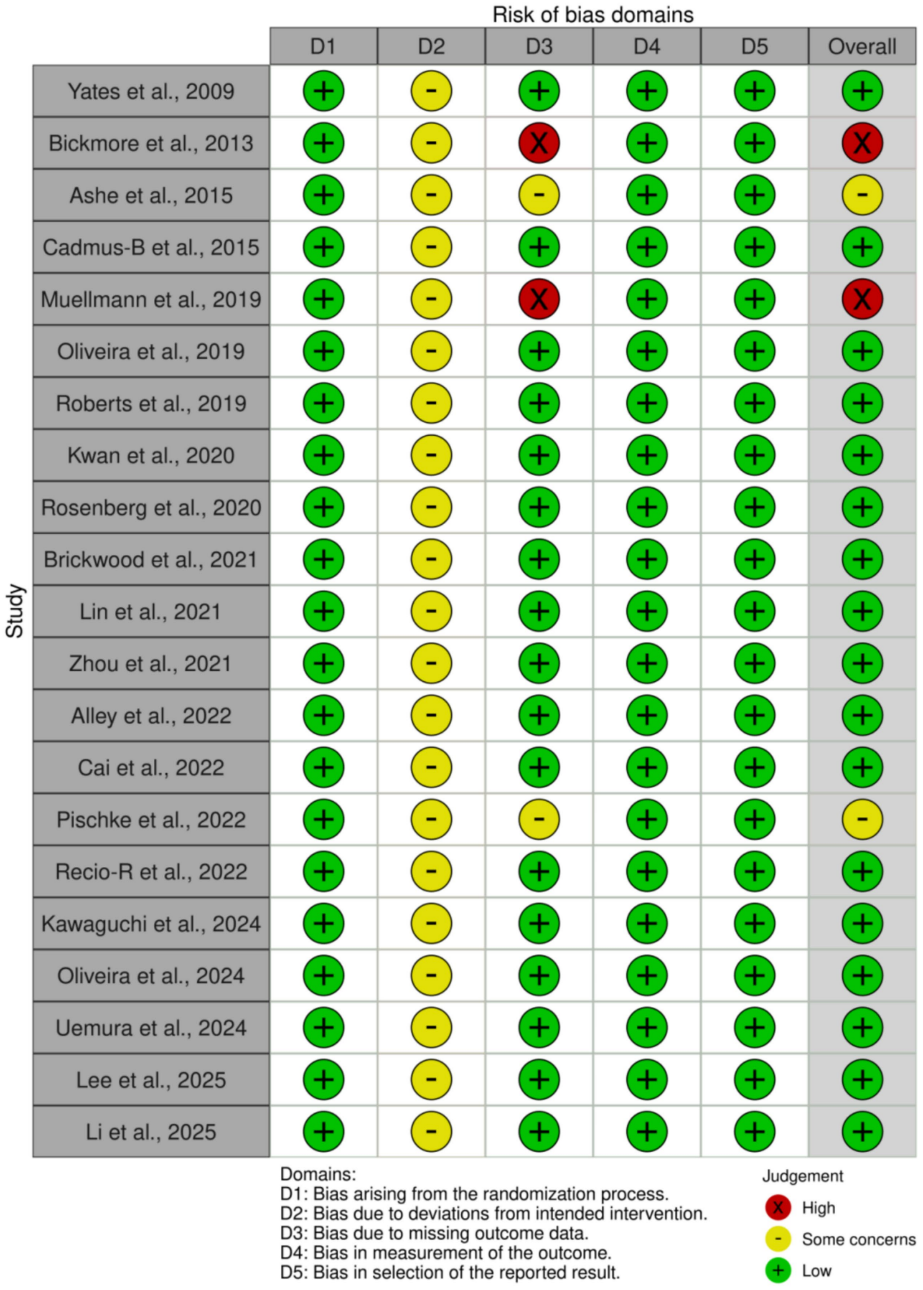
Risk of bias summary for the 21 included randomized controlled trials (RCTs) evaluating digital health interventions for older adults.

**Figure 3:**
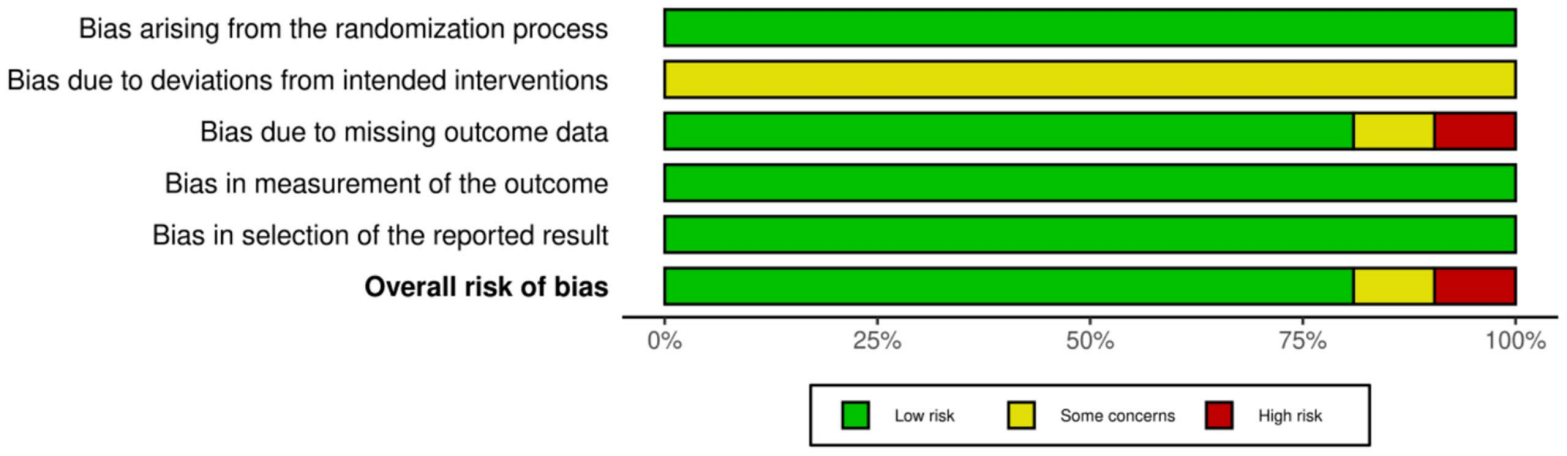
Risk of bias graph presented as cumulative percentages across the 21 included RCTs on digital health interventions for older adults.

This systematic review conducted a rigorous methodological quality evaluation on the 21 included publications, and the results indicated that most studies performed well in key assessment domains (Figures 2, 3). Assessments across the five dimensions (randomization process (D1), deviations from intended interventions (D2), missing outcome data (D3), measurement of the outcome (D4), and selection of the reported result (D5)) revealed that most studies were determined to have an overall “low risk,” reflecting the standardization of current digital health interventions in sports science research designs. Specifically, studies such as Alley et al. (46), Cai et al. (47), Li et al. (55), and Oliveira et al. (51) described random sequence generation and allocation concealment in detail, and evaded potential measurement bias (D4) arising from non-blinded designs through the use of objective monitoring tools such as accelerometers and pedometers. Furthermore, most studies ensured a low risk of bias within the selective reporting (D5) dimension due to comprehensive protocol registration and transparent results reporting. However, it must be cautiously noted that because of the overt nature of digital behavioral interventions, all included studies were judged as having “some concerns” in the dimension of deviations from intended interventions (D2). This objectively reflects the industry-wide limitation in physical activity interventions where participants and researchers cannot be blinded, meaning that potential interference from non-blinded designs on the intervention implementation process cannot be ruled out. Some studies were judged as having “some concerns” or “high risk” due to methodological limitations in specific domains. Bickmore et al. (35) and Muellmann et al. (38) were marked as high risk in the domain of missing outcome data (D3), primarily because of high participant attrition rates during the follow-up periods or imbalanced loss to follow-up proportions between groups, which could interfere with the veracity of the pooled effect sizes. Meanwhile, Ashe et al. (36) and Pischke et al. (48) were judged as having “some concerns” in the D3 dimension due to missing portions of raw data without explicitly stating the imputation methods. It is noteworthy that all included studies were judged as having “some concerns” in the dimension of deviations from intended interventions (D2), which objectively reflects the common industry characteristic in physical activity intervention research where double-blinding cannot be implemented for participants and researchers.

## 4 Results

### 4.1 Pooled results of outcome measures

#### 4.1.1 Daily steps

**Figure 4:**
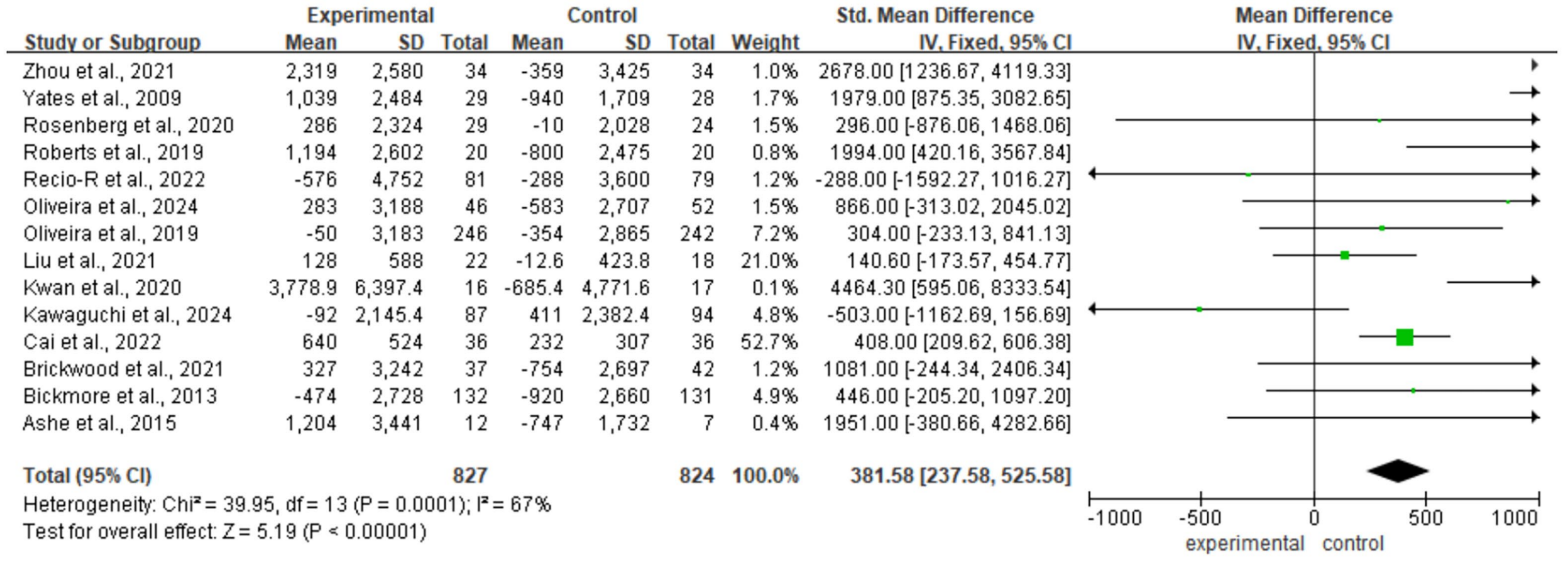
Meta-analysis forest plot of randomized controlled trials on the effects of digital health interventions based on objective monitoring on daily steps (steps/day) in older adults. The data in the forest plot utilize change-from-baseline scores (calculated as post-intervention values minus baseline values) to evaluate the net behavioral effect. Negative mean values observed in specific primary studies (e.g., Bickmore et al., (35) in the experimental group, and multiple studies in the control groups) strictly reflect a decrease in daily steps post-intervention relative to their respective baseline levels, rather than an absolute measurement of post-intervention steps.

Figure 4 illustrates the forest plot of digital health interventions (DHIs) for increasing daily steps in older adults. A total of 14 independent studies were included in this analysis, involving total sample sizes of 827 cases in the experimental group and 824 cases in the control group. The pooled overall effect size mean difference (MD) was 381.58, with a 95% confidence interval (CI) of [237.58, 525.58]. Regarding the heterogeneity test results, the chi2 statistic was 39.95, degrees of freedom (df) = 13, *p* = 0.0001, and *I*^2^ = 67%. The test for overall effect yielded a *Z*-statistic of 5.19, with a statistical significance level of *p* < 0.00001.

#### 4.1.2 MVPA

**Figure 5:**
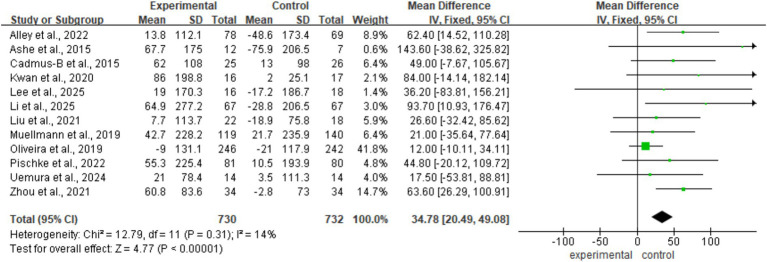
Meta-analysis forest plot of randomized controlled trials on the effects of digital health interventions based on objective monitoring on weekly moderate-to-vigorous physical activity (MVPA) duration (min/wk) in older adults. **Note:** The data in the forest plot utilize change-from-baseline scores (calculated as post-intervention values minus baseline values) to evaluate the net behavioral effect. Negative mean values observed in specific primary studies (e.g., Alley et al., (46) and Ashe et al., (36) in the control groups) strictly reflect a decrease in weekly moderate-to-vigorous physical activity duration post-intervention relative to their respective baseline levels, rather than an absolute measurement of post-intervention duration.

Figure 5 illustrates the forest plot of digital health interventions (DHIs) for increasing MVPA in older adults. A total of 12 independent studies were included in this analysis, involving 730 cases in the experimental group and 732 cases in the control group. The pooled overall effect size mean difference (MD) was 34.78, with a 95% confidence interval (CI) of [20.49, 49.08]. In the heterogeneity test, the chi2 statistic was 12.79, degrees of freedom (df) = 11, *p* = 0.31, and *I*^2^ = 14%. The test for overall effect yielded a *Z*-statistic of 4.77, with a statistical significance level of *p* < 0.00001.

### 4.2 Subgroup analysis

#### 4.2.1 Subgroup analysis for daily steps

The results of the subgroup analysis for daily steps (steps/day) are presented in detail in Table 2. Given the limited number of studies included under certain categories, the comparisons between groups in this section are exploratory analyses. Within the intervention duration dimension, the effect sizes for the short-term group (<12 weeks) and the long-term group (≥12 weeks) were 453.85 and 296.59, respectively, and the test for subgroup differences indicated no statistical significance (*p* = 0.29). Within the intervention tool dimension, the effect sizes for the mobile App group, wearable device group, and virtual coach group were 311.70, 961.22, and 971.71, respectively. The test for subgroup differences indicated a potential diverging trend (*p* = 0.02, *I*^2^ = 73.7%); however, considering that the wearable device and virtual coach groups contained only 4 and 3 studies, respectively, this significance cannot rule out the risk of spurious results caused by small-sample random errors. Within the interaction nature dimension, the human feedback group exhibited a strong effect trend (MD = 400.68, *p* < 0.00001), whereas the pooled effect size for the pure automation group was 229.29 [95% CI, −202.12, 660.71], and its test for overall effect did not demonstrate statistical significance (*p* = 0.30). Within the theoretical basis dimension, the effect sizes for the explicit theoretical framework group and the non-theory-driven group were 380.45 and 382.31, respectively, with a subgroup difference test of *p* = 0.99. Extrinsic heterogeneity was commonly present within each subgroup; except for the wearable device group (*I*^2^ = 41%), the I^2^ values for most subgroups ranged from 58 to 78% (see Supplementary Figures 1–4).

**Table 2:** Summary table of different subgroup characteristics regarding the effects of digital health interventions based on objective monitoring on daily steps (steps/day) in older adults.

Subgroup dimension	Subgroup classification	Number of Studies	Pooled Effect Size MD [95% CI]	Heterogeneity (I^2^)	Statistical Significance (*p*-value)
A. Intervention duration	<12 weeks	4	453.85 [257.97, 649.73]	69%	<0.00001
	≥12 weeks	10	296.59 [84.16, 509.01]	69%	0.006
B. Intervention tool	Mobile App/Mini-program	7	311.70 [159.36, 464.03]	70%	<0.0001
	Wearable device	4	961.22 [157.47, 1764.96]	41%	0.02
	Virtual coach/SMS	3	971.71 [443.40, 1500.01]	73%	0.0003
C. Interaction nature	Human feedback	7	400.68 [247.91, 553.44]	73%	<0.00001
	Pure automation/monitoring	7	229.29 [−202.12, 660.71]	66%	0.3
D. Theoretical basis	Explicit theoretical framework	8	380.45 [149.89, 611.01]	58%	0.001
	Non-theory-driven	6	382.31 [197.92, 566.70]	78%	<0.0001

#### 4.2.2 Subgroup analysis for MVPA

The results of the subgroup analysis for weekly moderate-to-vigorous physical activity (MVPA) duration are presented in detail in Table 3. Since the number of studies included under each subgroup category was relatively small (*n* = 4 or 6), the corresponding analysis results possess only academic reference value for hypothesis generation. Within the intervention duration dimension, the effect size for the le 12 weeks group was 51.26, which was higher than the 24.88 of the >12 weeks group, and the test for subgroup differences yielded *p* = 0.08. Within the population characteristics dimension, the effect size for the specific health risk population group was 57.15, which was numerically higher than the 23.55 of the general healthy older adults group, and the test for subgroup differences indicated statistical significance (*p* = 0.03); this result tends to serve as an exploratory hypothesis for future research. Within the intervention format dimension, the effect size for the pure software/web group was 54.96, and that for the software-hardware combination group was 32.41, with the test for subgroup differences yielding *p* = 0.34. Within the nature of the control group dimension, the effect size for the inactive control group was 51.44, and that for the active control group was 25.00, with the test for subgroup differences yielding *p* = 0.17. Inherent heterogeneity within each subgroup was relatively low; except for the intervention duration >12 weeks group (*I*^2^ = 25%) and the software-hardware combination group (*I*^2^ = 27%), the *I*^2^ values for most subgroups were below 20% or at 0% (see Supplementary Figures 5–8).

**Table 3:** Summary table of subgroup analysis results regarding the effects of digital health interventions based on objective monitoring on weekly moderate-to-vigorous physical activity (MVPA) duration (min/wk) in older adults.

Subgroup dimension	Subgroup classification	Number of Studies	Pooled effect Size MD [95% CI]	Heterogeneity (I^2^)	Statistical significance (*p*-value)
A. Intervention duration	≤12 weeks	6	51.26 [27.93, 74.60]	0%	<0.0001
	>12 weeks	6	24.88 [6.80, 42.97]	25%	0.007
B. Population characteristics	General community older adults	6	23.55 [6.03, 41.07]	12%	0.008
	Specific health risk population	6	57.15 [32.43, 81.87]	0%	<0.00001
C. Intervention format	Software-hardware combination group	8	32.41 [17.29, 47.52]	27%	<0.0001
	Pure software/web group	4	54.96 [10.94, 98.99]	0%	0.01
D. Nature of control group	Active control group	6	25.00 [7.90, 42.09]	17%	0.004
	Inactive control group	6	51.44 [18.05, 84.83]	0%	0.003

### 4.3 Reporting bias and robustness testing

**Figure 6:**
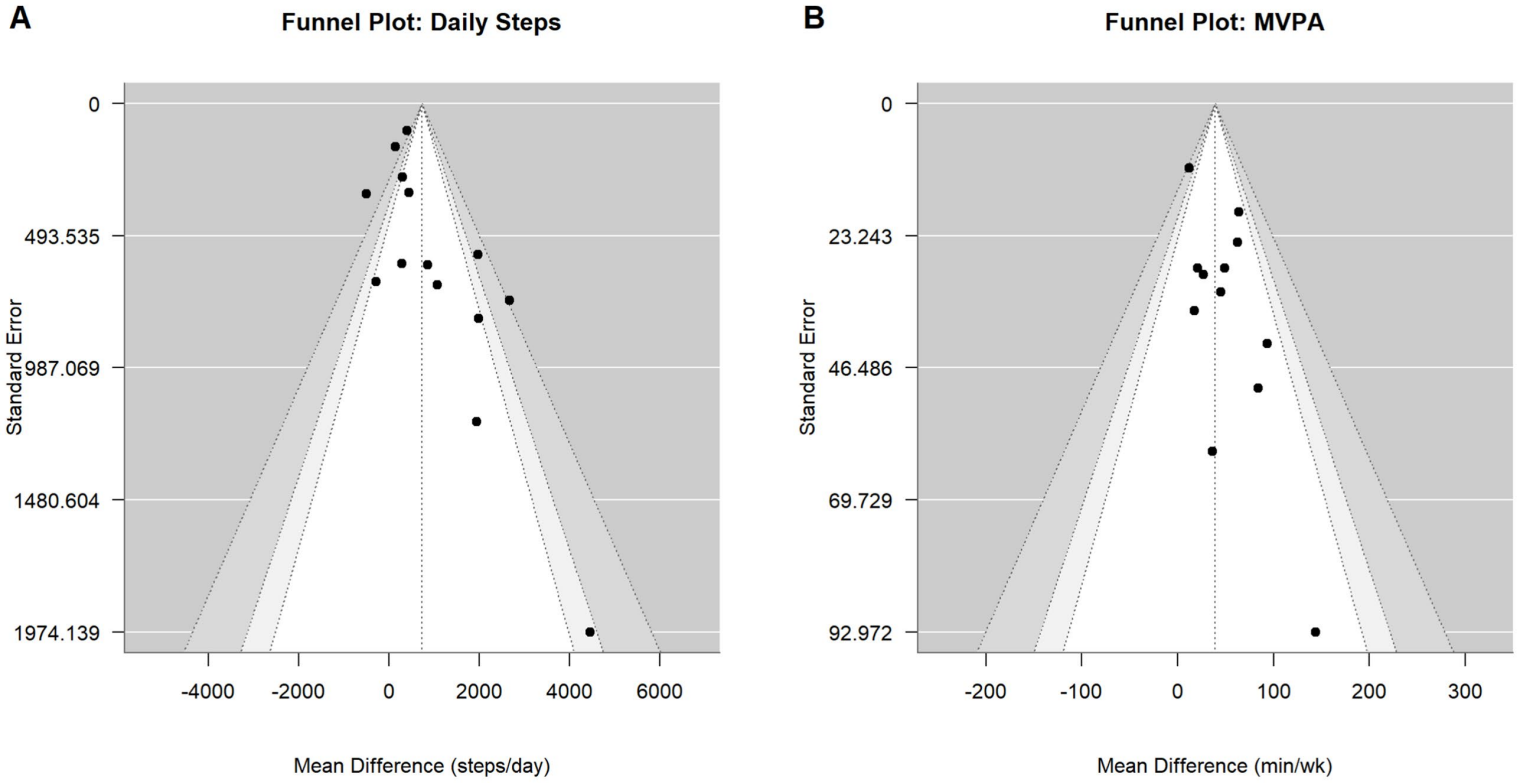
Funnel plots assessing publication bias: **(A)** funnel plot for daily steps; **(B)** funnel plot for moderate-to-vigorous physical activity (MVPA) duration.

Funnel plots were generated for daily steps and weekly moderate-to-vigorous physical activity (MVPA) duration in this study (Figure 6). In the funnel plot for daily steps, the scatter points were distributed relatively evenly on both sides of the midline; Egger’s test results showed *p* = 0.062, indicating that no significant publication bias was detected in a statistical sense. In contrast, the distribution of study scatter points for MVPA exhibited a distinct asymmetry. Quantitative evaluation showed that Egger’s test for MVPA was statistically significant (*t* = 2.47, *p* = 0.033), suggesting that the driving effect of digital interventions on this indicator was significantly confounded by small-study effects, and the original pooled effect size might entail a risk of being exaggerated.

**Figure 7:**
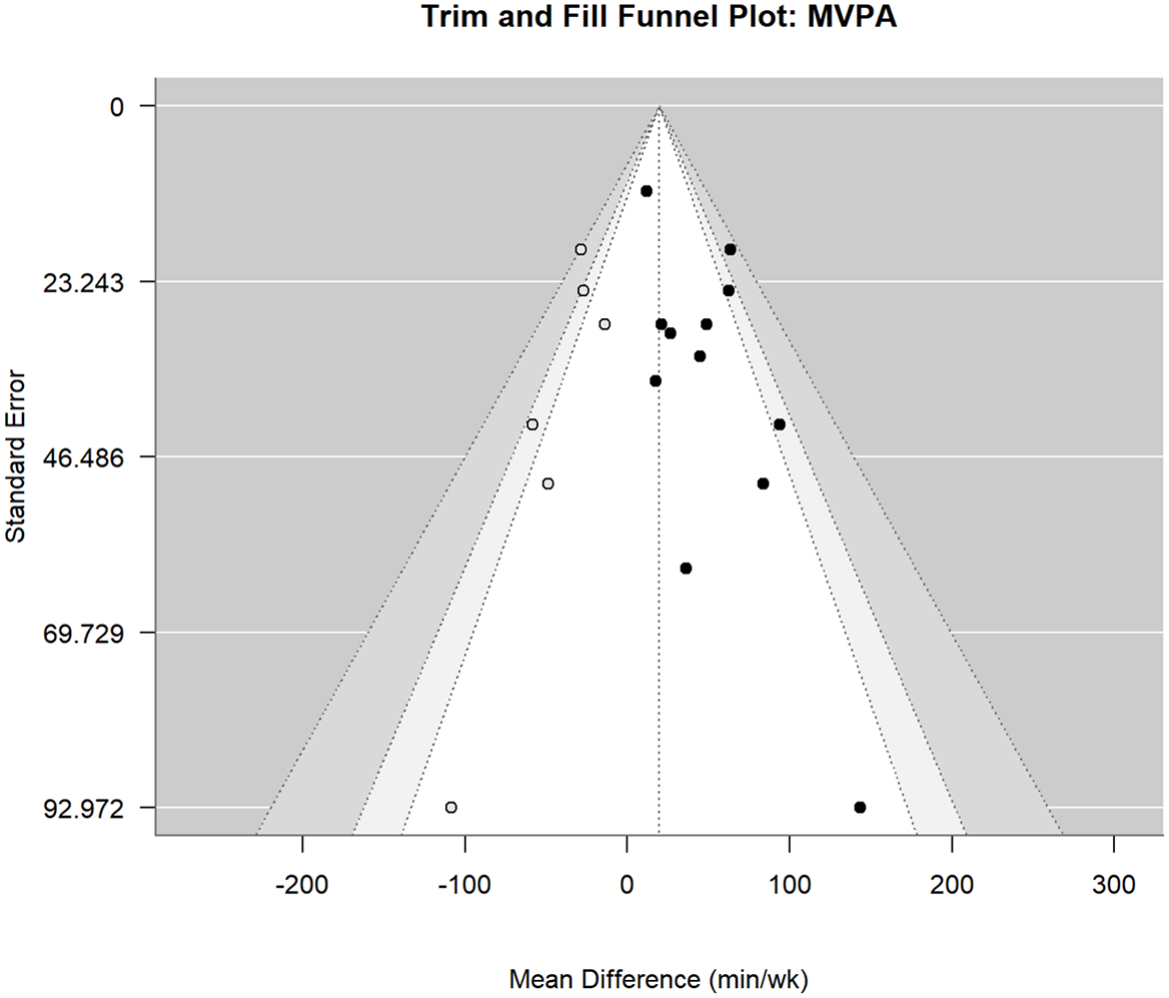
Trim-and-fill method on the effects of digital health interventions based on objective monitoring on weekly MVPA in older adults.

Given the potential risk of small-study effects for the MVPA indicator, this study utilized the trim-and-fill method to perform a robustness test. The trim-and-fill method identified 6 missing studies on the left side. After filling these potential studies, the simulated adjusted pooled effect size decreased from the original 34.78 to 19.70 [95% CI: −0.76, 40.16], and the statistical significance disappeared (*p* = 0.059). It must be noted that the trim-and-fill method is inherently an exploratory sensitivity analysis rather than a definitive causal diagnosis for the presence of publication bias. Given the heterogeneity across the included studies regarding technical carriers and specific delivery channels, the virtual estimates simulated by this algorithm through mathematical symmetry involve statistical uncertainty. Therefore, this adjusted result should only be interpreted as the lowest boundary of robustness for the pooled effect under the assumption of an extremely symmetrical distribution, suggesting that a cautious attitude should be maintained when interpreting the exact promotive effects of digital health interventions on MVPA in older adults (see Figure 7).

### 4.4 Evidence certainty evaluation

The study utilized the GRADE system to assess the certainty of evidence for each indicator reflecting the impact of digital health interventions on physical activity in older adults (see Table 4). The assessment results revealed that the certainty of evidence for weekly moderate-to-vigorous physical activity (MVPA) duration was rated at a “Low” level. Aside from being downgraded in the risk of bias dimension due to the common industry characteristic that double-blinding cannot be implemented in physical activity intervention research, this indicator was also downgraded in the publication bias dimension. Although its heterogeneity among original studies was low (*I*^2^ = 14%), Egger’s test identified a significant risk of publication bias (*p* = 0.033), and the trim-and-fill stress test showed that the adjusted pooled effect lost statistical significance (*p* = 0.059), indicating that the robustness of evidence for this indicator is weak and the original effect size is highly vulnerable to interference from small-study effects. Furthermore, the certainty of evidence for daily steps was strictly rated at a “Very Low” level. This indicator primarily faced a superposition of triple risks: first, in the inconsistency dimension, due to significant differences among the included studies regarding intervention tools (such as mobile Apps versus wearable devices), the studies exhibited a moderate-to-high heterogeneity (*I*^2^ = 67%, subgroup difference *p* = 0.02), resulting in a one-level downgrade; second, in the risk of bias dimension, affected by the high participant attrition rates in some studies and the inability to implement double-blinding, it was downgraded by another level; finally, funnel plot analysis showed an asymmetric distribution of scatter points with widely varying precision, indicating a potential risk of publication bias, resulting in an additional one-level downgrade. In summary, confounded by the triple interference of study inconsistency, risk of bias, and publication bias, the certainty of evidence for the effect of digital health interventions on increasing daily steps in older adults is at a very low level. Therefore, a high degree of caution must be maintained when interpreting and clinically translating the benefit results of this indicator.

**Table 4:** Summary table of evidence certainty grading based on the GRADE framework regarding the effects of digital health interventions based on objective monitoring on physical activity outcomes (daily steps, MVPA) in older adults.

Outcome Measure	Number of studies (k)	Sample size (*N*)	Effect size [95% CI]	Risk of Bias	Inconsistency	Indirectness	Imprecision	Publication Bias	Certainty
Daily steps (steps/day)	14	1,651	MD 381.58 [237.58, 525.58]	Serious	Serious	Not serious	Not serious	Serious	Very low ⊕◯◯◯
MVPA duration (min/week)	12	1,462	MD 34.78 [20.49, 49.08]	Serious	Not serious	Not serious	Not serious	Serious	Low ⊕ ⊕ ◯◯

## 5 Discussion

### 5.1 Summary of research findings

The results of this systematic review and meta-analysis suggest that digital health interventions (DHIs) based on objective monitoring demonstrate a divergence across outcome indicators in terms of improving physical activity levels among older adults. Regarding daily steps, the meta-analysis shows that the intervention groups increased by 381.58 steps compared with the control groups, and Egger’s test detected no significant publication bias (*p* = 0.062), suggesting that DHIs possess potential promotive benefits in increasing the total amount of daily activity in older adults. In contrast, the evidence for MVPA duration demonstrates a higher degree of instability; although the original pooled effect showed an increase of 34.78 min, Egger’s test identified a potential risk of small-study effects (*p* = 0.033). After performing the trim-and-fill sensitivity test and filling 6 studies, the pooled effect size decreased to 19.70 min and lost statistical significance (*p* = 0.059). This numerical fluctuation merely represents the boundary of robustness under an extreme assumption, suggesting that caution must be maintained when interpreting its exact overall effect. Subgroup analyses further suggest that the effects of DHIs are regulated by technical carriers and population characteristics, among which wearable devices and human feedback exhibit a trend toward better effect sizes for daily steps, while populations with specific health risks tend to display superior MVPA improvements compared with healthy older adults. Based on the GRADE system evaluation, the certainty of evidence for both outcome measures was rated at a “Low” level. Daily steps was downgraded due to high heterogeneity among studies (*I*^2^ = 67%) and a prediction interval that significantly crossed the line of null effect; meanwhile, the certainty of MVPA duration was downgraded due to its loss of robustness after the trim-and-fill adjustment, compounded by interference from the risk of bias.

### 5.2 Mechanisms and theoretical exploration of the effects of digital interventions based on objective monitoring on physical activity in older adults

The findings of this study demonstrate that digital health interventions (DHIs) based on objective monitoring can significantly improve the total amount of daily activity (daily steps) in older adults, but exhibit suboptimal robustness in driving exercise intensity (MVPA). This finding is partially consistent with the conclusions of Ran Li et al. (55) and Muellmann et al. (38), reinforcing that digital media can induce older adults to increase daily displacement through real-time step feedback. However, the conclusion of this study regarding the failure of MVPA under stress testing diverges from the robust benefits reported by Ashe et al. (36). The core reason for this inconsistency lies in the purity of the measurement tools: previous studies mostly utilized mixed subjective and objective data, which may mask the overestimation of high-intensity activity levels caused by self-report bias (56); whereas this study strictly limited inclusion to objective monitoring data, suggesting that the effect of a standalone technological tool to stably drive older adults across the physiological threshold of moderate-to-vigorous exercise warrants caution in the absence of real-time professional guidance.

The mechanism by which digital health interventions increase daily steps in older adults may be related to the real-time pairing of monitoring data with daily activities (26). Traditional exercise recommendations frequently compromise long-term adherence due to the lack of quantifiable standards, whereas digital tools utilize sensors such as accelerometers to transform physical displacement, which is originally difficult to perceive accurately, into intuitive numerical feedback (57). This display of results helps enhance participants’ perception of their own activity progress, thereby facilitating the achievement of exercise goals (58). Furthermore, the goal-setting function within DHIs breaks down complex exercise requirements into modular components, which may lower the action threshold for older adults to participate in physical exercise to a certain extent, and the achievement of daily goals can generate positive feedback for maintaining continuous exercise performance (59, 60). This behavioral promotive benefit has also been confirmed within a broader framework of social ecology and behavioral determinants. Evidence suggests that intervention measures targeting modifiable determinants, such as physical health and well-being of older adults, yield significantly superior ecological effects in promoting objectively measured physical activity (e.g., steps and MVPA) compared with programs relying solely on psychological or behavioral cognitive regulation (61); this indicates that the real-time physical data feedback inherent in digital tools achieves macro-level behavioral remodeling precisely by directly anchoring the core modifiable determinant of physical health perception. Concurrently, the results of this review based on strictly objective monitoring data further hint at the potential uncertainty of a standalone technological intervention in crossing the physiological threshold of moderate-to-vigorous exercise without professional guidance (62).

Subgroup analyses further elucidate the specific regulatory mechanisms of different intervention settings on effect sizes, involving multiple dimensions such as device interaction, human supervision, and population characteristics. In terms of device interaction, wearable devices are significantly superior to pure mobile Apps. This is attributable to the physical contact of hardware such as wristbands, which can implement passive reminders through vibration when users are sedentary; this method delivers a stronger passive reminder effect than Apps that require users to actively open their phones to check (26), thereby facilitating the reduction of sedentary time caused by oversight. Regarding human involvement, the human feedback groups performed significantly better than the pure automated monitoring groups (19), suggesting that for the older adult population, human participation can provide a necessary social supervision effect, and regular communication and encouragement help improve intervention adherence. From the perspective of population characteristics, the improvement effect on MVPA in populations with specific health risks is superior to that in healthy older adults, which may be explained by the fact that these populations with chronic diseases or health risks possess a more urgent demand for their own health, and the precise monitoring provided by digital tools tends to yield greater behavioral promotive benefits for the participants. Additionally, short-cycle interventions within 12 weeks exhibit better performance in increasing MVPA, which may be related to the high cooperation of older adults with new technologies during the initial stage of the intervention; as time progresses, the behavioral stimulus brought by external tools may gradually experience attenuation and blunting (18).

From a statistical perspective, this study highlights the potential volatility of quantitative evidence for digital interventions (63). Although Egger’s test for daily steps did not reach statistical significance (*p* = 0.062), demonstrating a certain degree of statistical stability, the high heterogeneity of I^2^ = 67% indicates that the intervention effects are profoundly regulated by the specific implementation environments (64). Furthermore, the MVPA indicator presents a potential risk of small-study effects (*p* = 0.033). Although its statistical significance altered after filling 6 studies using the trim-and-fill method (*p* = 0.059), this result merely represents the lowest boundary of robustness under the assumption of an extremely symmetrical distribution rather than a definitive attenuation of the intervention effect. This numerical fluctuation suggests that current research in this field is highly sensitive to small-study effects. Given the statistical uncertainty of the trim-and-fill algorithm itself under technical heterogeneity, it is impossible to definitively conclude that existing DHI protocols entail overly optimistic estimations; however, it reminds us to maintain a cautious attitude when evaluating the actual overall effect of digital feedback mechanisms in increasing exercise intensity (65). Future research still needs to further explore how to optimize digital feedback mechanisms to achieve the robust remodeling of physical activity patterns in older adults within complex real-world scenarios (66).

### 5.3 Research limitations

Although this study quantitatively evaluated the improvement effects of digital health interventions (DHIs) on objective physical activity indicators in older adults, several limitations still exist. First, the robustness of the core outcome measures is confounded by the dual interference of publication bias and high heterogeneity. The quantitative assessment of MVPA duration lost statistical significance under the trim-and-fill stress testing (adjusted *p* = 0.059), suggesting that its positive results are highly sensitive to small-sample studies. Concurrently, although the daily steps indicator demonstrated statistical significance, the high heterogeneity observed among studies attenuates the generalizability of the conclusions. Furthermore, in terms of methodological design, this review only searched publicly published English literature, and because of restrictions on the publication quality of peer review, gray literature databases and clinical trial registration platforms were not searched, which may have potentially exacerbated the risk of publication bias.

Second, the heterogeneity of participant population characteristics and intervention protocols restricts the extrapolation of the conclusions. The included participants ranged from healthy older adults to sub-healthy populations with specific clinical risks, meaning that the conclusions cannot be directly generalized to very old, frail individuals or older individuals with special physical load requirements. Simultaneously, the lack of uniformity in intervention protocols regarding the degree of device interaction (such as a standalone App versus a combination with a wristband) and the frequency of human interventions makes it difficult to provide standardized, universal exercise prescription recommendations.

Third, the non-uniformity in measurements and tool specifications constitutes methodological limitations. Although each study utilized “minutes/week” as the measurement unit for MVPA, different studies applied different physiological cut-points and epoch lengths when operating accelerometers (such as ActiGraph), which represents an important source of clinical heterogeneity. In addition, this review failed to fully align the precision differences between research-grade instruments (such as ActiGraph and activPAL) and commercial consumer-grade devices. Given that the older adult population commonly exhibits characteristics such as low walking speeds and irregular movement patterns, commercial devices may harbor hidden risks of missed recordings at low speeds, which may cause micro-level perturbations to the precision of the final pooled effect size.

Finally, the absence of blinding and the relatively short intervention duration exert potential interference on the strength of the evidence. Due to the inherent attributes of exercise behavior interventions, primary studies were universally unable to implement double-blinding, which may introduce unmonitored co-interventions such as peer encouragement, thereby overestimating participants’ adherence and exercise performance. Affected by this, according to the GRADE evaluation, the certainty of evidence for the core indicators was only at a “Low” or “Very Low” level. Coupled with the fact that existing studies mostly concentrated on short-to-medium-term interventions within 24 weeks, lacking long-term follow-ups after the cessation of interventions, whether DHIs can induce long-term physiological adaptations and behavioral habit remodeling still requires further verification by future large-sample, long-cycle studies.

### 5.4 Practical implications

The findings of this study provide an objective, evidence-based reference for health management and digital rehabilitation practices in older adults, with its application value primarily reflected across the following three levels: First, at the clinical and health management level, this study delineates the practical benefit boundaries of standalone digital interventions and highlights the upgrade pathway toward “hybrid interventions.” Although the meta-analysis demonstrates that DHIs can lead to a significant net increase of 381.58 daily steps in older adults, this dosage has not yet reached the recognized macro-threshold capable of inducing fundamental improvements in major clinical health outcomes (such as an increase of 1,000 steps) (46). This suggests that current standalone DHI protocols still exhibit limitations in driving older adults across the physical activity dosage required for substantial clinical benefits; coupled with the insufficient robustness of objectively measured MVPA in sensitivity testing, it is inappropriate to consider them as a standalone means to improve exercise intensity in practice. To this end, future clinical practices should deploy a hardware-software synergistic “hybrid intervention mode,” introducing human supervision and remote guidance to compensate for the deficiencies of pure automated monitoring. Concurrently, sports medicine practitioners should deeply align digital technology with the standard exercise prescription framework, namely the F. I. T. T. principle: in the frequency and time dimensions, automated behavioral triggering technologies of digital tools can be utilized to dynamically arouse sedentary behaviors based on real-time step counts, meticulously accumulating sporadic daily activity time; in the intensity dimension, smart devices should monitor heart rate zones or movement cadences in real time to provide precise biofeedback and safety warnings; in the type dimension, multi-modal interactive interfaces should be leveraged to modularly break down and personally deliver aerobic walking/running, resistance exercises, and balance training, thereby enhancing the operability of exercise prescriptions.

Second, at the precise intervention and policy formulation level, this study provides empirical evidence for the “precise implementation” of digital resource optimization and allocation. Subgroup analyses indicate that the improvement of activity volume by DHIs in populations with specific health risks tends to be superior to that in healthy older adults. Therefore, under circumstances of limited medical resources, policymakers may prioritize the allocation of digital resources to vulnerable populations with a higher risk of physiological degeneration. Furthermore, given that independent wearable devices exhibit a better trend in effect sizes compared with pure software interventions, industry standards should encourage the technological path of “hardware-software combination,” utilizing the immediate feedback and physical contact of hardware to assist in enhancing the intervention adherence of older adults.

Third, at the scientific research pathway level, this study outlines the scientific direction for future long-term behavioral evaluations of digital technologies. Given the high heterogeneity and potential risk of small-study effects exhibited by objective physical activity indicators, future studies urgently need to conduct large-sample, multi-center RCTs, focusing on examining the sustained effects of DHIs on long-term behavioral habit remodeling in older adults. Technological research and development should also transcend the scope of simple “data recording” to evolve toward closed-loop intervention systems that integrate intensity regulation, environmental perception, and real-time feedback, thereby enhancing the stability and long-term efficacy of intervention effects in complex, real-world scenarios.

## 6 Conclusion

The results of this systematic review and meta-analysis suggest that digital health interventions (DHIs) based on objective monitoring demonstrate a divergence across outcome indicators in terms of improving physical activity levels among community-dwelling older adults. The overall effect shows that DHIs help increase daily steps without significant publication bias detected. In contrast, although the original pooled effect for weekly moderate-to-vigorous physical activity (MVPA) duration was significant, it was confounded by potential publication bias and small-study effects, losing statistical significance after the trim-and-fill adjustment (*p* = 0.059) and demonstrating instability in its quantitative effect. Exploratory subgroup analyses suggest that wearable devices and human feedback for increasing steps, as well as specific health risk populations for improving MVPA, exhibit better trends in effect sizes. According to the GRADE evaluation, due to high extrinsic heterogeneity among studies, the absence of blinding control, and insufficient robustness after adjustment, the certainty of evidence for MVPA duration and daily steps was rated at a “Low” and “Very Low” level, respectively. Therefore, a cautious attitude should be maintained when generalizing and translating the current quantitative results. Future research is recommended to conduct large-sample, multi-center trials and explore transitioning from simple “data recording” to intelligent closed-loop intervention systems encompassing intensity regulation, environmental perception, and multi-dimensional feedback, thereby further confirming the long-term efficacy and stability of the intervention effects.

## Data Availability

The original contributions presented in the study are included in the article/Supplementary material, further inquiries can be directed to the corresponding author.
