# Supplementary material for: Effects of digital multimodal interventions on objectively measured physical activity in older adults: a systematic review and meta-analysis
Source: Front Public Health. 2026 Jun 25;14:1867281. doi: 10.3389/fpubh.2026.1867281 (PMC13345852; doi:10.3389/fpubh.2026.1867281)
Supplement: Supplementary file 1 [file Data_Sheet_1.DOCX]

**Supplementary Text 1 Search Strategies**

**Database: PubMed**

**Date of Search: April 12, 2026**

| **Step** | **Search Terms** | **Results** |
| --- | --- | --- |
| #1 | "Aged"[Mesh] OR "Aged, 80 and over"[Mesh] OR "Frail Elderly"[Mesh] | 3,861,217 |
| #2 | (older adult*[Tiab] OR elderly[Tiab] OR senior*[Tiab] OR geriatric*[Tiab] OR aging[Tiab] OR ageing[Tiab] OR late life[Tiab]) | 888,108 |
| #3 | #1 OR #2 | 4,299,955 |
| #4 | "Telemedicine"[Mesh] OR "Mobile Applications"[Mesh] OR "Smartphone"[Mesh] OR "Wearable Electronic Devices"[Mesh] OR "Fitness Trackers"[Mesh] OR "Monitoring, Physiologic"[Mesh] | 314,163 |
| #5 | (digital health[Tiab] OR mHealth[Tiab] OR eHealth[Tiab] OR telehealth[Tiab] OR mobile app*[Tiab] OR wearable*[Tiab] OR smartphone*[Tiab] OR pedometer*[Tiab] OR activity tracker*[Tiab] OR internet-based[Tiab] OR web-based[Tiab] OR online intervention*[Tiab] OR text messag*[Tiab] OR actigraph*[Tiab] OR accelerometer*[Tiab] OR sensor*[Tiab]) | 782,249 |
| #6 | #4 OR #5 | 1,018,382 |
| #7 | "Exercise"[Mesh] OR "Motor Activity"[Mesh] OR "Sedentary Behavior"[Mesh] OR "Walking"[Mesh] OR "Physical Functional Performance"[Mesh] | 419,878 |
| #8 | (physical activity[Tiab] OR exercise*[Tiab] OR walking[Tiab] OR daily steps[Tiab] OR step count*[Tiab] OR MVPA[Tiab] OR sedentary[Tiab] OR sitting time[Tiab] OR active lifestyle[Tiab] OR physical performance[Tiab]) | 670,197 |
| #9 | #7 OR #8 | 845,046 |
| #10 | "Randomized Controlled Trial"[Publication Type] OR "Controlled Clinical Trial"[Publication Type] | 750,033 |
| #11 | (randomized[Tiab] OR randomised[Tiab] OR RCT[Tiab] OR random allocation[Tiab] OR clinical trial*[Tiab] OR placebo[Tiab]) | 1,470,951 |
| #12 | #10 OR #11 | 1,719,576 |
| #13 | #3 AND #6 AND #9 AND #12 | 1894 |

**Database: Web of Science**

**Date of Search: April 12, 2026**

| **Step** | **Search Terms** | **Results** |
| --- | --- | --- |
| #1 | TS=("older adult*" OR elderly OR senior* OR geriatric* OR aging OR ageing OR "frail elderly" OR "late life") | 5,687,429 |
| #2 | TS=("digital health" OR mHealth OR eHealth OR telehealth OR "mobile app*" OR wearable* OR smartphone* OR pedometer* OR "activity tracker*" OR "internet-based" OR "web-based" OR "online intervention*" OR "text messag*" OR actigraph* OR accelerometer* OR sensor*) | 2,278,526 |
| #3 | TS=("physical activity" OR exercise* OR walking OR "daily steps" OR "step count*" OR MVPA OR sedentary OR "sitting time" OR "active lifestyle" OR "physical performance" OR "functional capacity") | 1,241,971 |
| #4 | TS=("randomized controlled trial" OR randomized OR randomised OR RCT OR "random allocation" OR "clinical trial*" OR "controlled clinical trial" OR placebo) | 1,902,956 |
| #5 | #1 AND #2 AND #3 AND #4 | 2265 |

**Database: Embase**

**Date of Search: April 12, 2026**

| **Step** | **Search Terms** | **Results** |
| --- | --- | --- |
| #1 | 'aged'/exp OR 'frail elderly'/exp OR 'very elderly'/exp | 4,717,069 |
| #2 | 'older adult*':ti,ab OR elderly:ti,ab OR senior*:ti,ab OR geriatric*:ti,ab OR aging:ti,ab OR ageing:ti,ab OR 'late life':ti,ab | 1,164,922 |
| #3 | #1 OR #2 | 5,229,231 |
| #4 | 'telemedicine'/exp OR 'mobile application'/exp OR 'smartphone'/exp OR 'wearable computer'/exp OR 'fitness tracker'/exp OR 'accelerometry'/exp OR 'sensor'/exp | 412,842 |
| #5 | 'digital health':ti,ab OR mhealth:ti,ab OR ehealth:ti,ab OR telehealth:ti,ab OR 'mobile app*':ti,ab OR wearable*:ti,ab OR smartphone*:ti,ab OR pedometer*:ti,ab OR 'activity tracker*':ti,ab OR 'internet-based':ti,ab OR 'web-based':ti,ab OR 'online intervention*':ti,ab OR 'text messag*':ti,ab OR actigraph*:ti,ab OR acceleromet*:ti,ab OR sensor*:ti,ab | 942,054 |
| #6 | #4 OR #5 | 1,114,297 |
| #7 | 'exercise'/exp OR 'motor activity'/exp OR 'sedentary behavior'/exp OR 'walking'/exp OR 'physical performance'/exp | 1,542,890 |
| #8 | 'physical activity':ti,ab OR exercise*:ti,ab OR walking:ti,ab OR 'daily steps':ti,ab OR 'step count*':ti,ab OR mvpa:ti,ab OR sedentary:ti,ab OR 'sitting time':ti,ab OR 'active lifestyle':ti,ab OR 'physical performance':ti,ab OR 'functional capacity':ti,ab | 957,579 |
| #9 | #7 OR #8 | 1,949,803 |
| #10 | 'randomized controlled trial'/exp OR 'randomization'/exp OR 'controlled clinical trial'/exp | 1,430,089 |
| #11 | randomized:ti,ab OR randomised:ti,ab OR rct:ti,ab OR 'random allocation':ti,ab OR 'clinical trial*':ti,ab OR placebo:ti,ab | 2,378,142 |
| #12 | #10 OR #11 | 2,803,905 |
| #13 | #3 AND #6 AND #9 AND #12 | 3781 |

**Database: The Cochrane Library**

**Date of Search: April 12, 2026**

| **Step** | **Search Terms** | **Results** |
| --- | --- | --- |
| #1 | [mh "Aged"] | 291573 |
| #2 | (elderly or "older adult*" or "older person*" or senior* or geriatric* or "aged 60" or "aged 65"):ti,ab,kw | 87291 |
| #3 | #1 OR #2 | 351350 |
| #4 | [mh "Telemedicine"] | 6149 |
| #5 | [mh "Internet-Based Intervention"] | 1100 |
| #6 | [mh "Mobile Applications"] | 2949 |
| #7 | [mh "Video Games"] | 1482 |
| #8 | ("digital health" or "e-health" or "ehealth" or "m-health" or "mhealth" or "mobile health" or "tele-health" or telehealth or "tele-medicine" or telemedicine or "smartphone" or "mobile app*" or "web-based" or internet* or "online intervention*" or "wearable*" or "digital intervention*" or "exergame*"):ti,ab,kw | 54961 |
| #9 | #4 OR #5 OR #6 OR #7 OR #8 | 57503 |
| #10 | [mh "Exercise"] | 42846 |
| #11 | [mh "Physical Analysis"] | 0 |
| #12 | [mh "Fitness Trackers"] | 277 |
| #13 | [mh "Actigraphy"] | 774 |
| #14 | ("physical activity" or "physical exercise" or "sedentary behavior" or "walking" or "step count*" or "daily steps" or "MVPA" or "acceleromet*" or "pedometer*" or "actigraph*" or "wearable device*" or "objective* monitor*" or "objective* measur*"):ti,ab,kw | 89427 |
| #15 | #10 OR #11 OR #12 OR #13 OR #14 | 113609 |
| #16 | #3 AND #9 AND #15 | 3263 |

**Database: CINAHL**

**Date of Search: April 12, 2026**

| **Step** | **Search Terms** | **Results** |
| --- | --- | --- |
| #1 | (MH "Aged+") OR (MH "Aged, 80 and Over") OR (MH "Frail Elderly") | 1,016,611 |
| #2 | TX (older adult* OR elderly OR senior* OR geriatric* OR aging OR ageing OR "late life") | 912,843 |
| #3 | #1 OR #2 | 1,631,680 |
| #4 | (MH "Telemedicine+") OR (MH "Mobile Applications") OR (MH "Wearable Technology") OR (MH "Monitoring, Physiologic+") | 162,833 |
| #5 | TX (digital health OR mHealth OR eHealth OR telehealth OR "mobile app*" OR wearable* OR smartphone* OR pedometer* OR "activity tracker*" OR "internet-based" OR "web-based" OR "online intervention*" OR "text messag*" OR actigraph* OR accelerometer* OR sensor*) | 359,982 |
| #6 | #4 OR #5 | 485,407 |
| #7 | (MH "Physical Activity+") OR (MH "Sedentary Behavior") OR (MH "Walking") OR (MH "Exercise+") OR (MH "Physical Performance") | 209,224 |
| #8 | TX (physical activity OR exercise* OR walking OR "daily steps" OR "step count*" OR MVPA OR sedentary OR "sitting time" OR "active lifestyle" OR "physical performance" OR "functional capacity") | 670,880 |
| #9 | #7 OR #8 | 682,516 |
| #10 | (MH "Randomized Controlled Trials") OR (MH "Clinical Trials+") | 377,067 |
| #11 | TX (randomized OR randomised OR RCT OR "random allocation" OR "clinical trial*" OR placebo) | 921,631 |
| #12 | #10 OR #11 | 926,747 |
| #13 | #3 AND #6 AND #9 AND #12 | 671 |
