# Supplementary material for: Effects of digital multimodal interventions on objectively measured physical activity in older adults: a systematic review and meta-analysis
Source: Front Public Health. 2026 Jun 25;14:1867281. doi: 10.3389/fpubh.2026.1867281 (PMC13345852; doi:10.3389/fpubh.2026.1867281)
Supplement: Supplementary file 2 [file Data_Sheet_2.DOCX]

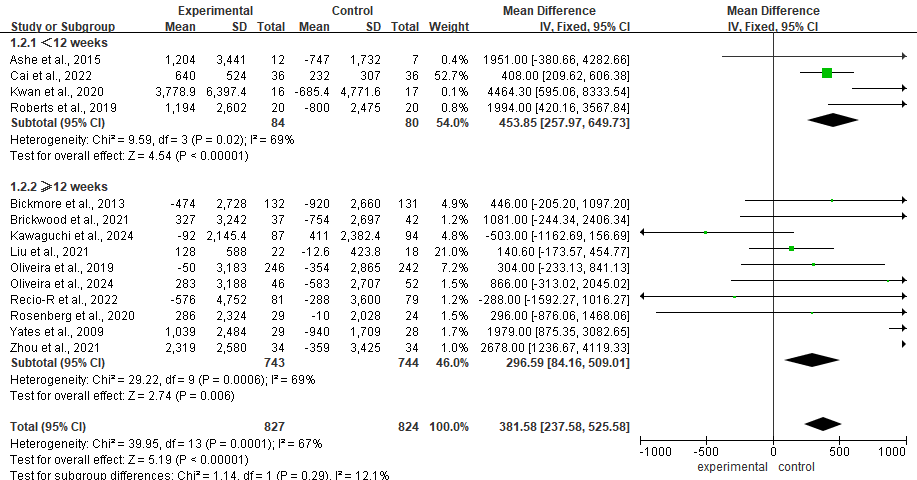


Supplementary Figure 1 Forest plot of subgroup analysis on the effects of different intervention durations on daily steps in older adults.


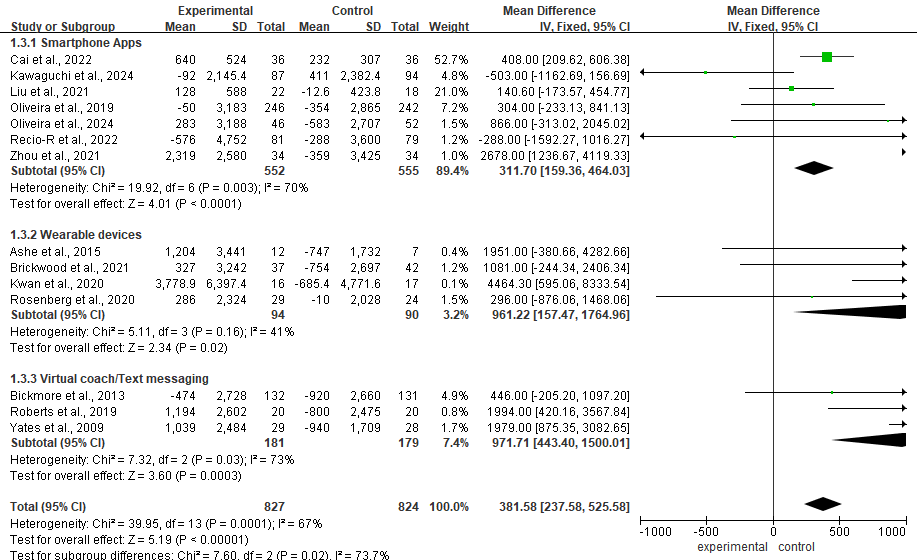


Supplementary Figure 2 Forest plot of subgroup analysis on the effects of different digital intervention modes on daily steps in older adults.


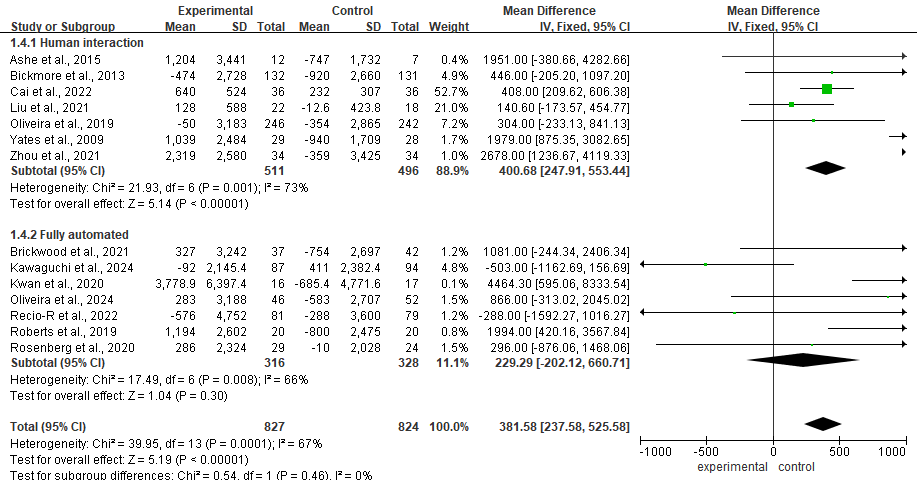


Supplementary Figure 3 Forest plot of subgroup analysis on the effects of different interaction types on daily steps in older adults.


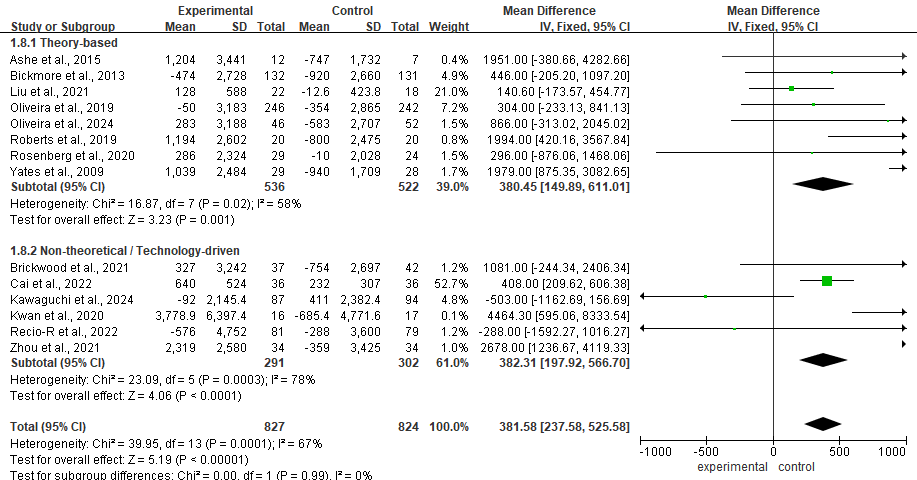


Supplementary Figure 4 Forest plot of subgroup analysis on the effects of theoretical frameworks on daily steps in older adults.


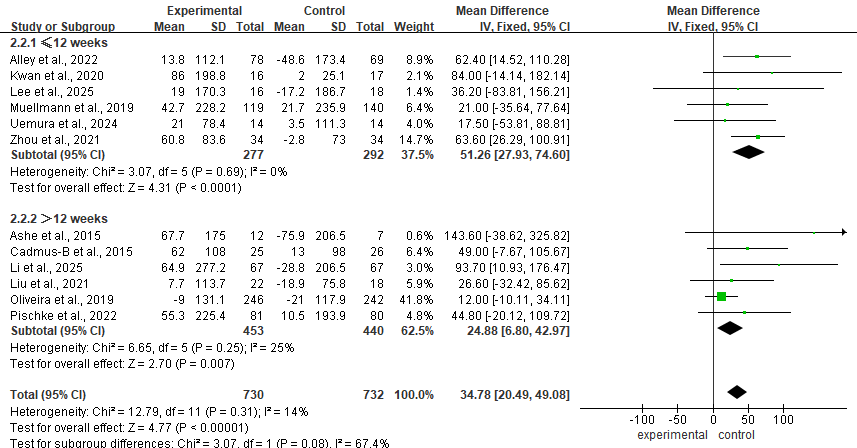


Supplementary Figure 5 Forest plot of subgroup analysis on the effects of different intervention durations on weekly MVPA time in older adults.


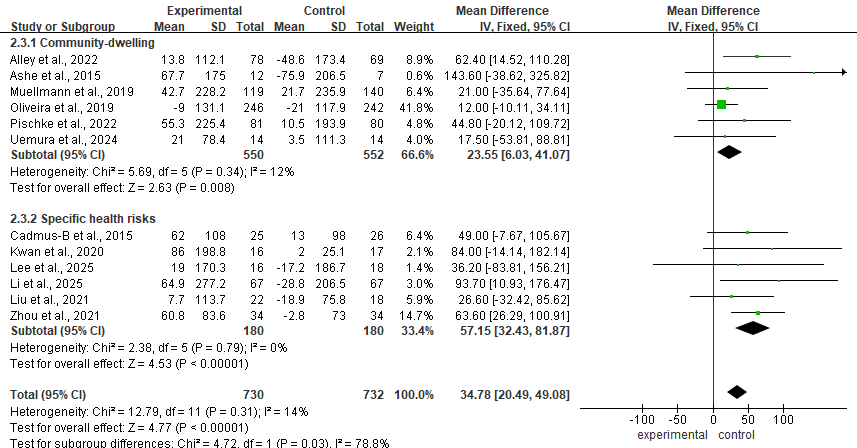


Supplementary Figure 6 Forest plot of subgroup analysis on the effects of different population characteristics on weekly MVPA time in older adults.


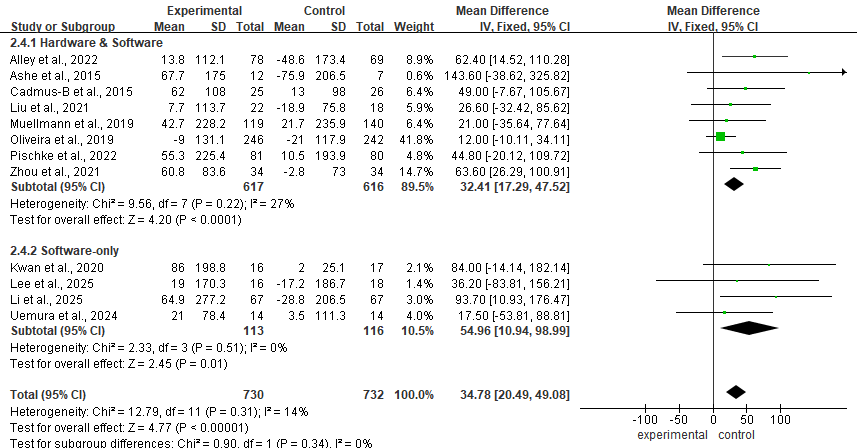


Supplementary Figure 7 Forest plot of subgroup analysis on the effects of different intervention forms on weekly MVPA time in older adults.


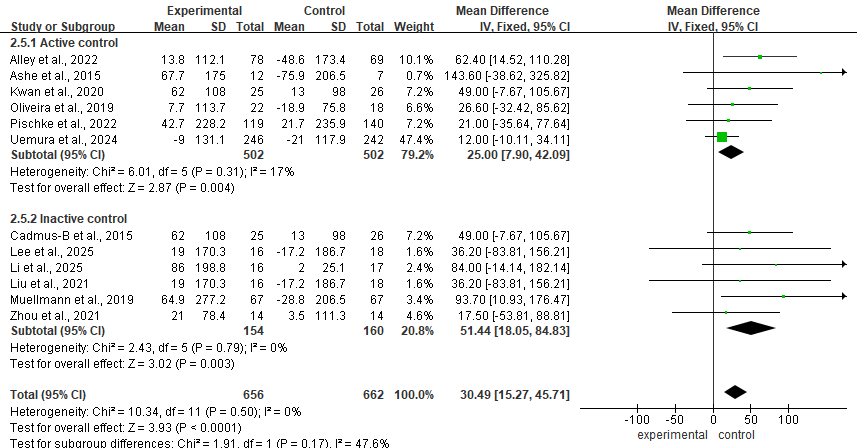


Supplementary Figure 8 Forest plot of subgroup analysis on the effects of different control group types on weekly MVPA time in older adults.
